# Draft Genome Sequence of the Obligate Halophilic *Bacillus* sp. Strain NSP22.2, Isolated from a Seasonal Salt Marsh of the Great Rann of Kutch, India

**DOI:** 10.1128/genomeA.01104-13

**Published:** 2013-12-19

**Authors:** Rinku Dey, Kamal Krishna Pal, Dharmesh Sherathia, Sejal Vanpariya, Ilaxi Patel, Trupti Dalsania, Kinjal Savsani, Bhoomika Sukhadiya, Mona Mandaliya, Manesh Thomas, Sucheta Ghorai, Rupal Rupapara, Priya Rawal

**Affiliations:** Microbiology Section, Directorate of Groundnut Research, Junagadh, Gujarat, India

## Abstract

Here, we report the 4.0-Mbp draft genome of an obligate halophile, *Bacillus* sp. strain NSP22.2, isolated from a seasonal salt marsh of the Great Rann of Kutch, India. To understand the mechanism(s) of obligate halophilism and to isolate the relevant gene(s), the genome of *Bacillus* sp. NSP22.2 was sequenced.

## GENOME ANNOUNCEMENT

The genomes of a number of species of *Bacillus*, isolated from the extremely saline environments of the Rann of Kutch, India, have been sequenced recently to understand the mechanisms of osmotolerance (1–4). *Bacillus* sp. strain NSP22.2 (16S rRNA GenBank accession no. JF802172), isolated from a seasonal salt marsh of the Great Rann of Kutch, India, grows optimally at a concentration of 7.0% NaCl (range, 5 to 20%) in growth medium, at pH 7.0 and 37°C. The genome of *Bacillus* sp. strain NSP22.2 was sequenced to understand its obligate and moderate halophilism and to isolate the relevant gene(s).

The Illumina HiSeq 2000 platform was used to sequence the genome of NSP22.2 at Macrogen, Inc., South Korea, through Sequencher Tech Pvt. Ltd., Ahmedabad, India. Originally, a total of 66,480,406 paired-end reads of 6,714,521,006 bases were generated, with a length of 72 nucleotides. After filtering (criteria, >90% of bases with a quality value [QV] of >20 for both ends) and the removal of PCR duplicates, 54,215,856 reads of 5,475,801,456 bases were obtained.

The draft genome was *de novo* assembled by SOAP*denovo* assembler version 1.05 (5), with genome coverage of about 1,200×. The long assembly, along with ordering of the contigs, was generated by OSLay (6). The draft assembly of NSP22.2 (G+C content of 43.99%) resulted in an approximate genome size of 4,000,934 bases, contained in 20 scaffolds (average scaffold, 200,047 bp; minimum, 1,155 bp; maximum, 1,400,974 bp) and 401 contigs (294 contigs of >500 bp) of 3,996,217 bp, with an average contig length of 9,965 bp (maximum, 80,648 bp; minimum, 84 bp). All the assembly data were deposited in the DDBJ/EMBL/GenBank nucleotide sequence database.

The draft genome sequence of NSP22.2 was further annotated using the RAST server (7), Glimmer 3 (8, 9), GeneMark (10), tRNAscan-SE (11), RNAmmer (12), and the KEGG database (13) for predicting subsystems, coding sequences (CDSs), tRNA and rRNA genes, KEGG pathways, and more.

Using the softwares, 4,251 CDSs were identified in the draft genome sequence of NSP22.2, with 53 RNA genes (51 tRNA and 2 rRNA) and 416 subsystems. Among the CDSs, 2,604 are not in a subsystem (967 nonhypothetical, 1,637 hypothetical), whereas 1,647 CDSs (1,560 nonhypothetical, 87 hypothetical) are in subsystems. RAST annotation also predicted the involvement of 105 genes in stress responses, including 27 genes involved in osmotic stress (2 in osmoregulation, 21 in choline and betaine uptake and betaine biosynthesis, and 4 in ectoine biosynthesis and regulation), 38 in oxidative stress (9 in protection from reactive oxygen species [ROS], 20 in oxidative stress, 2 in glutathione:nonredox reactions, 5 in redox-dependent regulation, 2 in glutaredoxins), 2 in cold shock, 15 in heat shock, 1 in detoxification, and 22 in no subcategory. Also, 7 genes involved in potassium homeostasis, 10 in glycerol and glycerol-3-phosphate uptake and utilization, 8 in mannitol utilization, 314 in biosynthesis and utilization of amino acid and derivatives, and 447 in utilization of carbohydrates, as well as other genes, were identified. Moreover, 1,976 genes were mapped in different pathways of KEGG (K00003 to K16697).

Work is in progress to unravel the mechanisms of obligate halophilism and adaptation in *Bacillus* sp. NSP22.2 and to identify the gene(s) relevant to osmotolerance.

### Nucleotide sequence accession numbers.

This whole-genome shotgun project has been deposited at DDBJ/EMBL/GenBank under the accession no. AVCV00000000. The version described in this paper is version AVCV01000000.
